# Spectroscopic and Mechanical Properties of a New Generation of Bulk Fill Composites

**DOI:** 10.3389/fphys.2016.00652

**Published:** 2016-12-27

**Authors:** Riccardo Monterubbianesi, Giovanna Orsini, Giorgio Tosi, Carla Conti, Vito Librando, Maurizio Procaccini, Angelo Putignano

**Affiliations:** ^1^Department of Clinical Sciences and Stomatology, Polytechnic University of MarcheAncona, Italy; ^2^Department of Materials, Environmental Science and Urban Planning, Polytechnic University of MarcheAncona, Italy; ^3^Department of Chemical Sciences, University of CataniaCatania, Italy

**Keywords:** bulk fill resin composites, degree of conversion, surface microhardness, curing lamps, spectroscopy

## Abstract

**Objectives:** The aims of this study were to *in vitro* evaluate the degree of conversion and the microhardness properties of five bulk fill resin composites; in addition, the performance of two curing lamps, used for composites polymerization, was also analyzed.

**Materials and Methods:** The following five resin-based bulk fill composites were tested: SureFil SDR®, Fill Up!™, Filtek™, SonicFill™, and SonicFill2™. Samples of 4 mm in thickness were prepared using Teflon molds filled in one increment and light-polymerized using two LED power units. Ten samples for each composite were cured using Elipar S10 and 10 using Demi Ultra. Additional samples of SonicFill2, (3 and 5 mm-thick) were also tested. The degree of conversion (DC) was determined by Raman spectroscopy, while the Vickers microhardness (VMH) was evaluated using a microhardness tester. The experimental evaluation was carried out on top and bottom sides, immediately after curing (t0), and, on bottom, after 24 h (t24). Two-ways analysis of variance was applied to evaluate DC and VMH-values. In all analyses, the level of significance was set at *p* < 0.05.

**Results:** All bulk fill resin composites recorded satisfactory DCs on top and bottom sides. At t0, the top of SDR and SonicFill2 showed the highest DCs-values (85.56 ± 9.52 and 85.47 ± 1.90, respectively), when cured using Elipar S10; using Demi Ultra, SonicFill2 showed the highest DCs-values (90.53 ± 2.18). At t0, the highest DCs-values of bottom sides were recorded by SDR (84.64 ± 11.68), when cured using Elipar S10, and Filtek (81.52 ± 4.14), using Demi Ultra. On top sides, Demi Ultra lamp showed significant higher DCs compared to the Elipar S10 (*p* < 0.05). SonicFill2 reached suitable DCs also on bottom of 5 mm-thick samples. At t0, VMH-values ranged between 24.4 and 69.18 for Elipar S10, and between 26.5 and 67.3 for Demi Ultra. Using both lamps, the lowest VMH-values were shown by SDR, while the highest values by SonicFill2. At t24, all DC and VMH values significantly increased.

**Conclusions:** Differences in DC and VMH among materials are suggested to be material and curing lamp dependent. Even at t0, the three high viscosity bulk composites showed higher VMH than the flowable or dual curing composites.

## Introduction

In nowadays dentistry, resin-based composites have been and are widely used for dental restorations, even if with some disadvantages: shrinkage, shrinkage stress, micro cracks (in the dental structure or in the resin material), debonding, and secondary caries (Ilie et al., 2007; Ilie and Hickel, 2011; Tantbirojn et al., 2011; El-Saftya et al., 2012; Van Ende et al., 2012; Czasch and Ilie, 2013).

In the time, the multi-layer technique tried to reduce these drawbacks: the fact to place composites in dental cavities, using thin increments of 2 mm or less, allowed a good penetration of the light-curing, thus lowering the shrinkage stress (El-Saftya et al., 2012). Nevertheless, this procedure might afford air bubbles, no linkage between layers, troubles during composites placing, especially in the posterior areas, in which it is sometimes difficult to fill the deep cavities and it is often required a long chair time (Abbas et al., 2003; Sarrett, 2005).

To solve these problems, the manufacturers introduced bulk fill resin composites, consisting in new chemical monomers and fillers with an enhancement of their translucency and, consequently, with the potentiality of obtaining an optimal degree of conversion (DC), even in the bottom of the cavities, where it is more difficult to reach high DC. It has been demonstrated that there are several factors affecting the mechanical properties of a resin composite: chemical composition, amount of emitted radiation, distance from the tip of the light source, and photo-activation mode (Da Silva et al., 2008b). Recent improvements in nanotechnology have led to an optimal filler content of this new generation composites, by the addition of free nanosized spherical particles and clusters, which can act as a single unit, thus significantly improving their mechanical properties, also after finishing and polishing procedures (Beun et al., 2007; Jung et al., 2007; Czasch and Ilie, 2013). Furthermore, the benefit of these materials consists in the fact that they can be cured to a maximal increment thickness of 4–6 mm with a limited shrinkage, due to their high translucency, thus allowing the clinicians to rapidly fill the cavity, shortening the chair time (Van Ende et al., 2012). However, even if the first generation bulk filling materials (introduced by Dentsply with a product called SDR) presented a limited shrinkage stress, they showed unsatisfactory mechanical properties, due to the low percentage of fillers, thus requiring the use of a conventional resin composite, acting as an enamel-top capping layer (Campodonico et al., 2011; Ilie and Hickel, 2011; Van Dijken and Pallesen, 2014).

These limitations were improved with the introduction of high viscosity bulk fill composites, which can fill up the occlusal area in a unique step (as single bulk increment), cured and hence sculpted, without the need of an additional top capping layer. Indeed, innovative composites have been recently available on the market, such as SonicFill, which, for instance, uses the sonic energy to decrease viscosity; once the sonic energy has been removed, the resin composite gradually returns to the starting high viscosity status, assuring good mechanical properties (Ahmad, 2013).

The recent large use of high viscosity bulk fill composites have been due to the following positive factors: simplified procedures, increase of the filler percentage, high depth of cure, acceptable translucency, negligible shrinkage stress after polymerization, and satisfactory cavity adaptation (Ahmad, 2013).

As every modern resin-based system, also bulk fill composites necessitate the light curing process to be polymerized. It is noteworthy that clinicians have the tendency to overestimate the properties of bulk fill resin composites, giving an incorrect evaluation of the volumetric shrinkage, shrinkage stress, and DC. Moreover, other factors such as modulus of elasticity, rate of polymerization, polymerization kinetics, initiator chemistry, gel point, type of filler and monomer, development and intensity of the curing stress have to be taken into account (Ferracane et al., 2014). In fact, even if bulk filling restorations were recommended also in cavities up to 4 mm deep, it turned out that many practitioners are still improperly doubtful on their suitability in the clinical field (Czasch and Ilie, 2013).

The clinical performance of posterior resin-based composite restorations can be strongly affected by various parameters as DC and surface hardness. A high DC can determine good mechanical properties, chemical stability and longevity of the restoration (Da Silva et al., 2008a); it has been measured using different methods, such as Raman, NIR (near-infrared) and MIR (middle-infrared) spectroscopies, in order to evaluate changes of aliphatic/aromatic double bond ratio. A satisfactory polymerization degree is essential for the success of the restoration and can require even more than 24 h (Yoon et al., 2002; Miyazaki et al., 2003; Conti et al., 2005).

The surface hardness (measured as microhardness, by means of Vickers or Knoops tests) is defined as the resistance to permanent indentation or penetration on time, and it has to be evaluated in finishing and polishing phases, or when the resin composites are placed on large areas of masticatory force (Galvao et al., 2013; Tarle et al., 2015).

In order to evaluate the acceptable values of DC and surface microhardness, it is worldwide accepted to refer to the International Standard Test ISO 4049-2009, introduced for dental polymer-based restorative materials. In fact, to consider the suitability of a dental restoration (Flury et al., 2012), it is mandatory to take into account the determination of the depth of cure of a dental composite, referring to both DC and Vickers microhardness (VMH) as fundamental key tests (Flury et al., 2012; Leprince et al., 2012).

The primary aim of this study was to analyse the mechanical and spectroscopic properties of one low viscosity bulk fill composite (SDR), one medium viscosity/dual curing bulk fill composite (Fill Up!), and three high viscosity bulk fill composites (Filtek, SonicFill, SonicFill2), by means of DC and VMH measures. In addition, the potential correlation between DC and VMH was also evaluated.

The secondary aim was to evaluate the performance of two curing lamps in the photo-polymerization process of the different composite samples.

The null hypotheses were: (1) DC values and VMH values do not significantly change within the tested bulk fill composites and there is a correlation between DC and VMH; (2) there are no difference in the performance of the two tested curing lamps.

## Materials and methods

Five commercial (shade 3) bulk fill composites for posterior restorations were tested (Table 1):

1. SureFil® SDR® (from now on called SDR), a low viscosity flowable composite (Smart Dentin Replacement, Dentsply Caulk, Milford, DE, USA), which shows, in the manufacturer instructions, to need a final top capping layer.
2. Fill Up!™(Coltène Whaledent AG, Altstätten, Switzerland), a medium-viscosity/dual curing bulk composite, which requires one step increment and no top capping layer, as shown in the manufacturer instructions.
3. Filtek™ Bulk Fill Posterior Restorative (3M ESPE, St Paul MN, USA) a high viscosity bulk composite, which requires one step increment, no top capping layer as shown in the manufacturer instructions.
4. SonicFill™ (Kerr Corp. Orange, CA, USA), and
5. SonicFill2™ (Kerr Corp. Orange, CA, USA). Both SonicFill systems combine a flowable resin composite with an universal resin composite by using a hand piece which enables sonic activation. As shown by the manufacturer instructions, SonicFill2™ presents improved mechanical properties compared to SonicFill™.

**Table 1:** **Chemical composition of the tested bulk fill composites**.

**Materials**	**Manufacturer**	**Type**	**Composition**
SureFil® SDR®	Dentsply Caulk	Bulk-fill flowable composite	modified UDMA, TEGDMA, EBPDMA, pigment, photoinitiator, barium and strontium alumino-fluoro-silicate glasses, Silicon Dioxide—Amorphous, Strontium. Aluminosilicate Glass. Filler load: 68 wt%; 45 vol%.
Fill up!™	Coltène/Whaledent AG	Dual curing bulk composite	TMPTMA, UDMA, bis-GMA, TEGDMA, dibenzoyl peroxide; benzoyl peroxide, Zinc oxide coated. Filler load: 65 wt%; 49 vol%.
Filtek™ bulk fill posterior restorative	3M/ ESPE, St. Paul, MN, USA	Bulk-fill paste composite	Bis-GMA, bis-EMA, UDMA, zirconia, Filler load:76.5 wt%, 58.4 vol%.
SonicFill™	Kerr Corporation, CA, USA	Bulk-fill paste composite activated	Resin: EBADMA, BisphenolA-bis-(2-hydroxy-3-mehacryloxypropyl) ether, TEGDMA, 3-trimethoxysilylpropyl methacrylate, SiO_2_, Glass, oxide, chemicals. Filler load: 83.5 wt%; 83 vol%.
SonicFill2™	Kerr Corporation, CA, USA	Bulk-fill paste composite activated	Poly(oxy-1,2-ethanediyl), α,α′-[(1-methylethylidene)di-4, 1-phenylene]bis[ω-[(2- methyl-1-oxo-2-propen-1-yl)oxy]-Not available. 2,2′-ethylenedioxydiethyl dimethacrylate.
			Filler load: 81.3% wt % unreported.

*Bis-GMA, bisphenol-Aglycidyldimethacrylate; bis-EMA, ethoxylated bisphenol-A-dimethacrylate; UDMA, urethane dimethacrylate; TMPTMA, trimethylolpropane Trimethacrylate; TEGDMA, triethylene glycol dimethacrylate; TEGDMA, triethylene glycol dimethacrylate; EBADMA, ethoxylated bisphenol-A dimethacrylate; EBPDMA, ethoxylated Bis-GMA; SiO_2_, silicon dioxide; wt%, weight percentage; vol%, volume percentage*.

The two light-curing lamps used for the polymerization of resin-composite samples presented the following technical details. Elipar S10 (3M ESPE) had an energy output 1200 mW/cm^2^ and spectrum between 430 and 480 nm; on the other hand, Demi Ultra (Kerr Corp.) had variable energy output from 1100 to 1330 mW/cm^2^ and spectrum between 450 and 470 nm, the energy intensity gradually changing from 1100 until 1330 mW/cm^2^.

### Degree of conversion (DC)

To evaluate DC, homemade Teflon cylinders (of 4 mm in height and 6 mm of internal diameter) were used; for each curing lamp, 10 disk-shaped specimens of each of five resin composites were obtained and photo-polymerized in bulk for 20 s. During the photo-polymerization, to exclude oxygen contamination, each sample was covered with a mylar strip on both surfaces. It has to be noted that the sample mass simulates the amount of resin composite usually used to fill up a dental cavity of recurrent dimensions. All the samples were measured on top side (top) and on bottom side (bottom), immediately once cured (t0), and, only on bottom, after 24 h (t24), too. In addition, for SonicFill2, the DC measurements were performed also in 10 3 mm-thick and 10 5 mm-thick samples, and finally, a last measurement was performed after 240 h (t240) only in SonifFill2 4 mm-thick samples.

**Figure 1:**
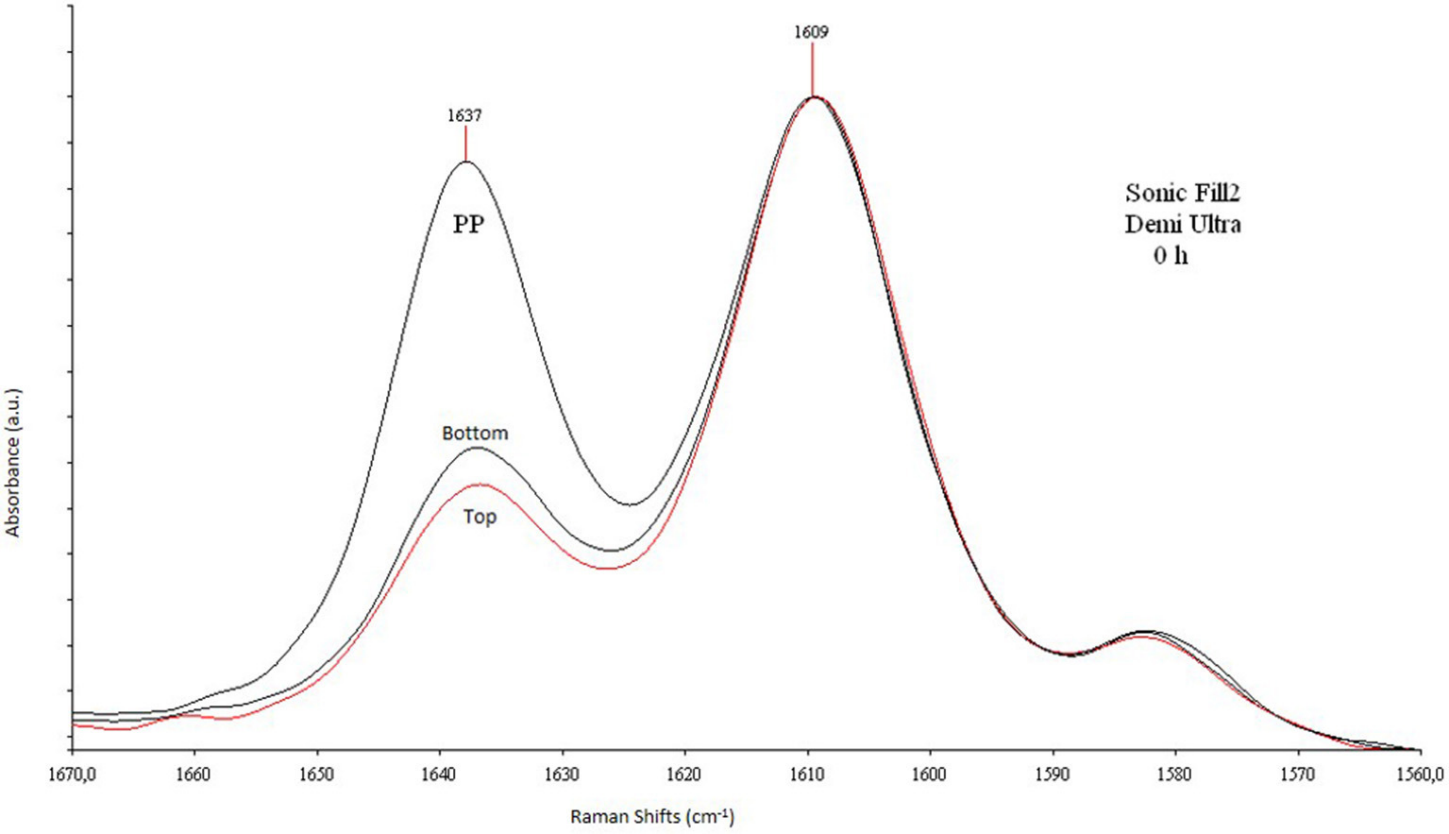
**Raman spectra between the reaction and reference bands**. Raman spectra in the region 1670–1560 cm^−1^ of SonicFill2, 4 mm-thick, cured with Demi Ultra.

A DXR FT Raman spectrometer (Thermo Fischer Scientific, Zug, Switzerland) was used to obtain the Raman spectrum of bulk composites. In Raman determinations, DC was evaluated by comparing the ratio of the alkene carbon-carbon double bond (1638 cm^−1^, reaction band B), which was formed during the polymerization, with the one of the aromatic benzene ring (1610 cm^−1^), whose intensity does not change during the polymerization (reference band A). To evaluate the DC, calibration curves were plotted assuming that the ratio A/B on top of the no cured material may represent the 0% of polymerization, while the same ratio on the top at t24, may be taken as 100% of polymerization. For this reason, the top DC-values will not be described in the results. Figure 1 shows a typical Raman spectrum with the decrease of olefinic C = C mode at 1635 cm^−1^ (reaction band) during the curing of both surfaces.

### Vickers microhardness

Vickers microhardness was determined with Leitz Micro-Hardness (Wetzal GMBH, Wetzlar, Germany) tester on the same samples used for the DC measurements. The method consisted in indenting the sample by a diamond indenter with the form of a right pyramid. In our case, a 50 g load was applied for 15 s. Once the load was removed, it was possible to evaluate (by using a microscope) the corresponding average value of the two indentation diagonals, to get the area of the sloping surface and, hence, to determine the corresponding hardness value.

The measurements of VMH were achieved at t0 and at t24 on the irradiated top surface of the samples. Three measurements were made for each sample: on the middle, on 0.15 mm and on 0.3 mm from the center. Calculations were made by using a computer software (Hardness-Course Vickers/Brinell/Rockwell copyright IBS 2012 version 10.4.4) (Fleming et al., 2008; Roberts et al., 2009; Nagi et al., 2015).

### Statistical analyses of DC and VMH data

Statistical analyses were performed by means of R Project for Statistical Computing 3.3.0 (https://www.r-project.org/) and Microsoft Excel 2013. Normality of data distribution and homogeneity of group variances were verified by Kolmogorov-Smirnov test and Levene test, respectively. Differences of DC and VMH-values among groups and at different time intervals were evaluated by two-ways analyses of variance (Two-Ways ANOVA). The Tukey test was applied for *post-hoc* comparisons. In all analyses, the level of significance was set at *p* < 0.05.

## Results

Descriptive statistics of DC-values are reported in Table 2. Two different curing lamps were used in this study, Elipar S10 and Demi Ultra. At t0, they recorded statistically significant different DC-values between top and bottom sides (*p* < 0.05).

**Table 2:** **Degree of Conversion (DC)**.

**Composites**	***N***	**t0 top**		**t0 bottom**		**t24 bottom**	
		**DC%**	**SD%**	**DC%**	**SD%**	**DC%**	**SD%**
**ELIPAR S10**							
SDR	10	85.56	9.52	84.64	11.68	93.17	8.04
FILL UP!	10	82.79	3.20	65.03	6.57	94.71	7.96
FILTEK	10	78.27	3.95	74.32	7.30	92.20	9.01
SONICFILL	10	70.40	10.66	68.12	6.29	78.76	8.13
SONICFILL2	10	85.47	1.90	77.01	8.47	78.62	9.23
**DEMI ULTRA**							
SDR	10	84.93	2.26	75.67	2.20	91.94	10.02
FILL UP!	10	88.75	3.89	65.45	11.36	75.45	14.05
FILTEK	10	86.54	2.15	81.52	4.14	98.84	4.17
SONICFILL	10	82.30	6.03	78.78	5.90	90.95	8.92
SONICFILL2	10	90.53	2.18	75.44	3.53	80.25	7.32

*N, number of samples; DC, Degree of Conversion; SD, Standard Deviation*.

*Mean of DC-values with Standard Deviation at t0, on top and bottom, together with t24 bottom values of samples cured using Elipar S10, and Demi Ultra lamps, respectively*.

On top sides, at t0, DC-values of tested composites ranged between 70.40 and 85.56% using Elipar S10. On the other hand, using Demi Ultra lamp, top DC-values at t0 ranged between 82.30 and 90.53%. At t0, top DC-values obtained by Demi Ultra were significantly higher than the ones obtained using Elipar S10 (*p* < 0.05). SDR and SonicFill2 had higher DC-values than the other composites cured using Elipar S10; SonicFill2 recorded the highest DC-values when cured using Demi Ultra.

**Figure 2:**
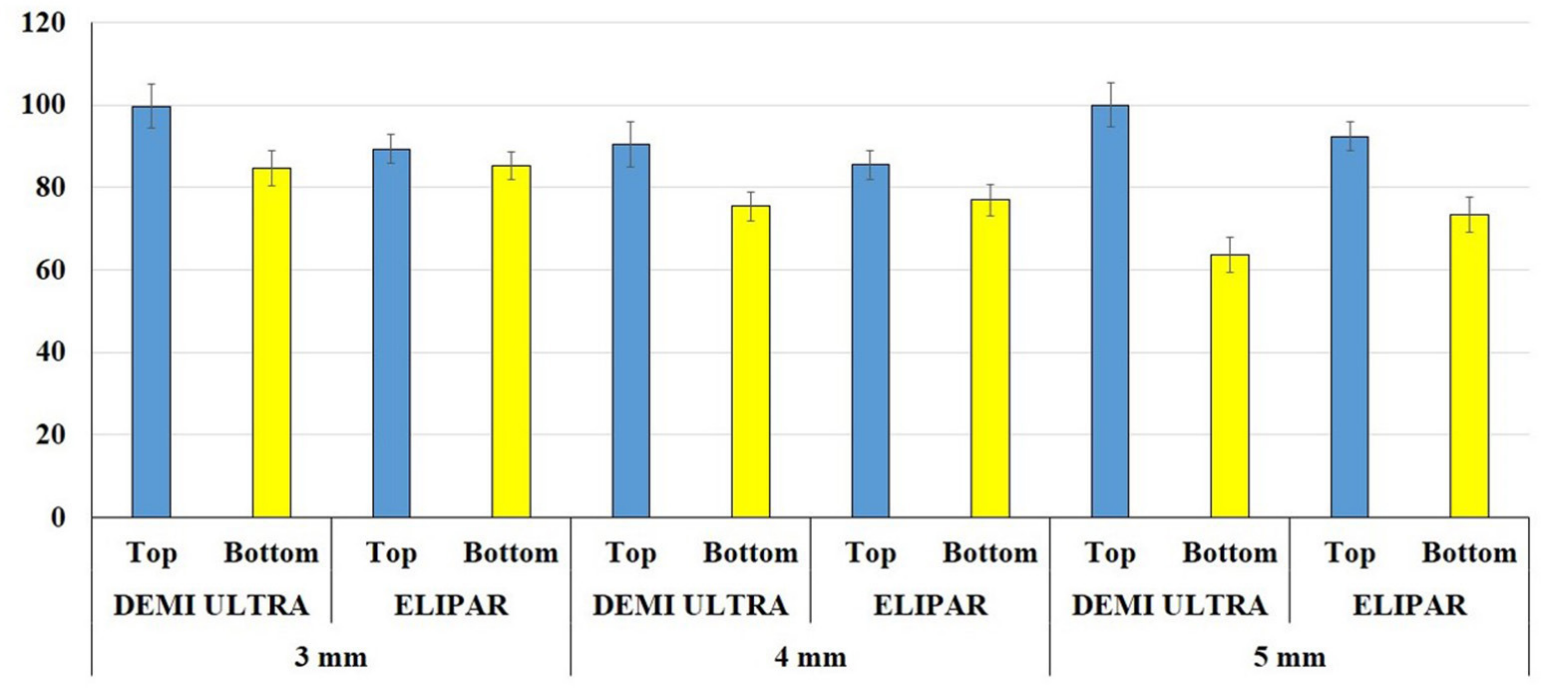
**Degree of Conversion (CD) comparison between top and bottom at different time**. Mean of DC top and bottom values at t0 of SonicFill2 in 3, 4, and 5 mm-thick samples, cured using Demi Ultra and Elipar S10 lamps.

On bottom side, at t0, DC-values ranged between 65.03 and 84.64%, using Elipar S10. On the other hand, using Demi Ultra, they ranged between 65.45 and 81.52%. At t0, the highest bottom DC-values were recorded by SDR cured by Elipar S10 and Filtek cured by Demi Ultra (*p* < 0.05). After 24 h, all DC-values significantly increased (*p* < 0.05). Both at t0 and t24, the values recorded by the two curing lamps were not statistically different (*p* > 0.05). An additional DC evaluation was performed on SonicFill2 samples of different thickness (3 and 5 mm-thick) at t0, using both lamps. Figure 2 shows means of DC-values on top side of 3, 4, and 5 mm-thick samples, that were not statistically different (*p* > 0.05). On the other hand, on bottom side, the mean DC-values in the 3 mm (84.2%) and 5-thick samples (68.7%) were statistically different (*p* < 0.05). The last evaluation of SoniFill2 samples (4 mm-thick) after 240 h showed no significant difference of DC-values on top between t24 and t240 (*p* > 0.05).

**Figure 3:**
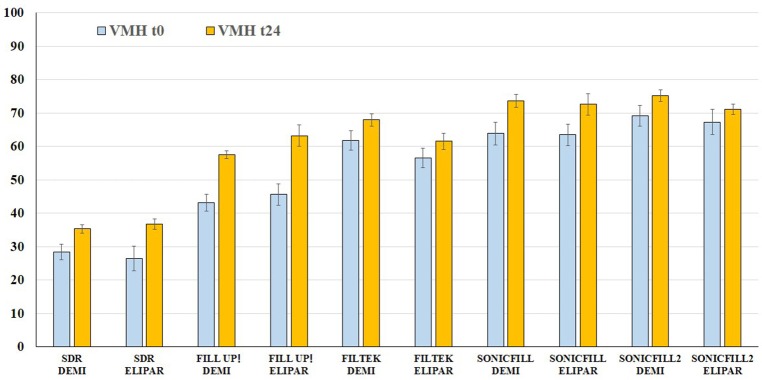
**Vickers microhardness (VMH) comparison on top at different time intervals**. Mean and Standard Deviation of VMH-values of the five composites at t0 and t24 using Demi Ultra (DEMI) and Elipar S10 (ELIPAR) lamps.

The VMH-values recorded on the top of the different specimens by using the two curing lamps were statistically different (Figure 3). Demi Ultra showed higher VMH-values than Elipar S10 (*p* < 0.05). The mean of VMH measurements (from t0 to t24) showed the following significant increase (*p* < 0.05): 51.9 at t0 vs. 61.04 at t24 (using Elipar S10); 53.28 at t0 vs. 61.91 at t24 (using Demi Ultra). SonicFill2 had the highest VMH-values (*p* < 0.05): being 69.18 ± 3.15 at t0, using Demi Ultra, and 75.2 ± 1.69 at t24; using Elipar S10, 67.3 ± 3.7 at t0 and 71.12 ± 1.52, at t24. On the other hand, SDR showed the lowest VMH-values (*p* < 0.05): being 28.4 ± 2.34 at t0 and 35.28 ± 1.2 at t24, using Demi Ultra; using Elipar S10, 26.5 ± 3.71 at t0 and 36.73 ± 1.48 at 24.

Finally, Pearson's Test indicated that there is no correlation between DC and VMH, using both curing lamps (0.24 for Demi Ultra, 0.016 for Elipar S10).

## Discussion

Nowadays, it is becoming of growing tendency to use bulk fill resin composite materials because of their simplified procedures for filling in a single increment posterior restorations compared to the multi-increments techniques required by conventional resin composites. Indeed, manufacturers and recent scientific reports demonstrate that the main advantages of this restorative procedure consist in an increased depth of cure and a low polymerization shrinkage (Ilie et al., 2013; Leprince et al., 2014). Dental restorative composites polymerize to a certain depth, depending on the light beam penetration of the curing lamp inside the mass (Leloup et al., 2002), and a suitable polymerization of the whole composite mass remains one of the main important factors influencing the clinical success (Czasch and Ilie, 2013). In fact, high DC-values are important to assess optimal physical, mechanical and biological properties of resin composites; on the other hand, when there is not an optimal DC incomplete polymerization (unreacted—dangerous—monomer), marginal microleakage, discoloration, decrease of bonding strength and low mechanical properties can occur (Yap et al., 2004; Kusgoz et al., 2011; Alonso et al., 2013; Galvao et al., 2013). For a clinically acceptable restoration, some authors indicate that the DC-value should reach at least the 55% of it, however, even if resin-based dental materials are properly cured, they generally exhibit a significant amount of unreacted monomers (Soares et al., 2007; Galvao et al., 2013). High values of DC, up to 60%, can be due to improvements in the resin matrix (flexibility and viscosity of the starting monomer), to the composition and size of fillers and to the irradiance intensity (Dickens et al., 2003; Turssi et al., 2005).

In this study, in order to evaluate the DC-values of the tested bulk fill composites, which are based on the use of prepolymerized resins, it was assumed that the ratio 1635/1610 (described above) of the starting material, before the curing, may represent the 0% of polymerization, whereas the ratio of 100% of polymerization is assumed at t24 on the samples top sides (Ferracane, 1995; Blackham et al., 2009). For this reason, at t24, the DC-values on the top were not evaluated. Indeed, due to the overlapping of the reaction bands at 1635 cm^−1^ at t24 with the one at 240 h (e.g., SonicFill2), it has been hypothesized that after 24 h the polymerization process can be reasonably concluded. Therefore, these two reference points allow us to establish the evolution in time of the polymerization process: it was between t0 and t24, or between 0 and 100%. Raman spectroscopic evaluation shows that different DC-values are recorded between top and bottom at t0 for all composites, using the two curing lamps (*p* < 0.05). On top and at t0, Demi Ultra seems to cure better than Elipar S10 (*p* < 0.05), while on bottom and at t0, the two curing lamps are not statistically different to cure SDR and Filtek, which show the highest DC-values using Elipar S10 and Demi Ultra, respectively. After 24 h, bottom DC-values increase using both Elipar S10 and Demi Ultra, with no significant differences between the two curing lamps: once again SDR and Filtek show the best performance. In agreement with literature reports, all 4 mm-thick tested samples show high DC-values, mainly for t0 bottom determinations (Goracci et al., 2014; Leprince et al., 2014; Marovic et al., 2015). In the case of SDR, the high DC-values can be due to the high fluidity and transparency of this resin, even if, it is noteworthy again to remark, that a flowable resin composite, like SDR, needs an additional hardening top layer. Literature data concerning SonicFill and SDR evidence some discordance and, in general, lower DC-values than our data, mainly for t0 bottom determinations (57.9 and 50.3%, respectively, when using Demi Ultra lamp, with an output of 1100 mW/cm^2^; see Goracci et al., 2014). Indeed some reports show that SDR DC-values on top ranged from the 77% (t0), using a light unit output of 1200 mW/cm^2^ (Guimaraes et al., 2013), to the 67%, reported by other authors (Van Ende et al., 2013; Marovic et al., 2015). Among the high viscosity samples, SonicFill2 shows the highest DC-value with both curing lamps (at t0, on top), being Demi Ultra the best unit to polymerize it. However, both curing lamps result in a satisfactory performance, being Demi Ultra slightly superior than Elipar S10 in top surfaces curing (*p* < 0.05).

As mentioned above, DC-values of SonicFill2 have been determined also on 3 and 5 mm-thick samples at t0 (Figure 2). The bottom DC mean values of these samples at different thickness are statistically different, thus suggesting that the thickness of samples could condition the DC. However, it is noteworthy to underline that acceptable DC-values are registered even on the bottom side of 5 mm-thick samples.

Finally, the fact that there is no difference in DC SonicFill 2 values between t24 and t240 means that a satisfactory degree of polymerization can be reached after 24 h, in agreement with other literature reports (Ferracane, 1995; Blackham et al., 2009).

Hardness and microhardness measure the resistance to plastic deformation and indicates the resistance to indentation under functional stresses: a high value can be indicative of the ease of finishing and polishing of a restoration (Rahiotis et al., 2004; David et al., 2007). It has already been reported that hardness values may be related to the DC of carbon double bonds of a resin composite; even if with some divergence, it has been shown that a conversion around 90% of the resin may correspond to a bottom/top VMH ratio of 80% (Bouschlicher et al., 2004). Moreover, the microhardness of a resin composite depends also on the thickness: an increase in thickness causes a microhardness lowering.

In recent years, modulator interacting with camphorquinone (as in the case of the dual-curing composite Fill Up!), transparency enhancements and/or technological up grades (as in the case of high viscosity bulk fill composites) have been added to the resin composites, thus resulting in satisfactory microhardness values even in 4 mm-thick samples. Indeed, some authors reported that high viscosity bulk fill composites might exhibit high microhardness values because of the high percentage of fillers content (Barabanti et al., 2015; Nagi et al., 2015). Figure 3 reports mean and SD of VMH top values at t0 and t24, using the two curing lamps. As expected, after 24 h, all samples show significant VMH increases (*p* < 0.05). Among the five materials, the lowest VMH mean value is shown by SDR, while a considerable increase is found for the medium viscosity dual curing Fill Up! (57 and 63 vs. 51 VMH, as claimed by the manufacturer Coltene); noteworthy is that at t0, SonicFill2 shows the highest VMH-value, using both curing lamps.

**Figure 4:**
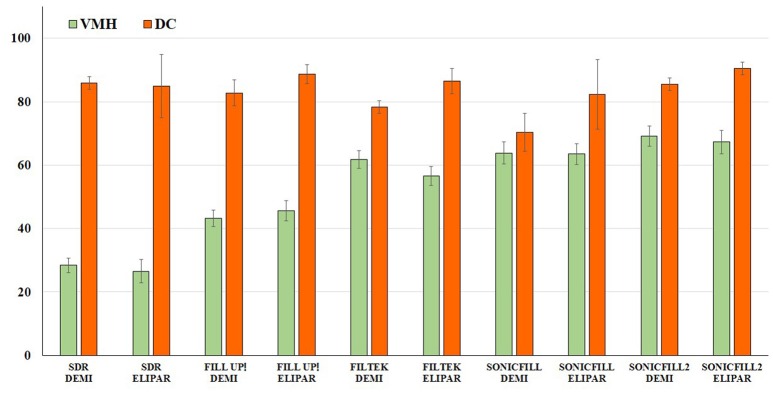
**Correlation between Vickers microhardness (VMH) and Degree of Conversion (DC)**. Comparison between mean VMH and DC-values (top t0) of the samples cured using Demi Ultra (DEMI) and Elipar S10 (ELIPAR) lamps.

Several studies have tried to find a correlation between the DC and VMH for some authors no correlation may be drawn, while, for others, a negative trend occurs between the two parameters (Ferracane, 1985; De Wald and Ferracane, 1987; Santos et al., 2007; Da Silva et al., 2008a). In our case, the lack of any correlation is evident (Pearson's correlation coefficient: 0.24 for Demi Ultra, −0.016 for Elipar S10), meaning that, even if high values of two single parameters may point out a satisfactory dental curing, a linear correlation is lacking (Figure 4). This fact may be due to the different contents and viscosity of the five composites (Table 1).

In the attempt to analyse the behavior of a reasonable range of new bulk fill dental composites, a significant number of samples have been evaluated in the present work, however, other experiments are needed to make definitive remarks and, therefore, future analyses will aim to continue to analyse newly introduced materials. In definitive, differences in DC and VMH-values between our study and previous literature reports can be ascribed to improvements of fillers (composition and particles size distribution, percentage of filler load), of the resin matrix (monomer type and its chemical structure), and also to the enhanced performance of the new curing lamps. Indeed, it is important to underline the great impact that nanotechnology has produced in terms of development and progressing of dental materials science. Therefore, the present study can be considered relevant since there are no previous reports evaluating all the five bulk fill resin composites, using vibrational techniques, such as Raman spectroscopy, and also because this is the first time in which the performance of two new generation curing lamps have been analyzed.

## Conclusions

In the light of the presented results, the two null hypotheses can be rejected: firstly, because there are differences in DC and VMH within the tested bulk fill composites, and no correlation can be drawn between DC and VMH; secondly, because the behavior of the two curing units is slightly different. Within the limits of the current study, it can be concluded that the five resin composites recorded satisfactory polymerization degree on both top and bottom sides.

To summarize, the flowable SDR shows high DC-values with both lamps, while, among high viscosity samples, SonicFill systems (and especially SonicFill2), combining the advantages of a flowable dental material with a universal resin composite, by using sonic activation, demonstrate excellent DC-values. In particular, SonicFill2 showed the highest DC-value using both curing lamps (at t0, on top), and an acceptable DC, even in case of 5 mm-thick samples.

Significant differences in VMH-values among the five tested materials are found: the lowest value has been evidenced by the flowable SDR, reinforcing the need of a top capping layer, while, in agreement with the literature, an appreciable increase has been found for the dual curing Fill Up!. Among the high viscosity resin composites, a satisfactory performance has been demonstrated by SonicFill and, mainly, by SonicFill2. Both DC and VMH determinations appear clinically significant to make a prevision of the future performances of restorations. The use of medium and, mainly, high viscosity bulk fill materials may also be important to avoid a further capping application. Moreover, this study can be useful to increase the knowledge of clinicians in understanding the curing performance of the tested lamps.

Our upcoming aim will be to increase the number of the tested dental composites, as well as to further study by means of vibrational techniques both dental and composites surfaces after finishing and polishing.

## Author contributions

RM contributed to the research protocol, performed the experiments as part of his Ph.D. project, contributed to the results analyses and writing. GO contributed to the idea, the research protocol, and the writing of the present manuscript. GT contributed to the correct developing of the research idea, the writing, the technical background of the entire manuscript. CC contributed to the technical idea and support all experimental phases. VL contributed to the Raman facilities use and data interpretation, and to the discussion finalization. MP contributed to the idea and the updated results organization of the manuscript. AP contributed to the idea, the findings interpretation, and the editing of the manuscript.

## Funding

Dental materials were gently donated from the manufacturers.

### Conflict of interest statement

The authors declare that the research was conducted in the absence of any commercial or financial relationships that could be construed as a potential conflict of interest. The reviewer ED and handling Editor declared their shared affiliation, and the handling Editor states that the process nevertheless met the standards of a fair and objective review.
